# Supplementary material for: Epistasis arises from shifting the rate-limiting step during enzyme evolution of a β-lactamase
Source: Nat Catal. 2024 Feb 23;7(5):499–509. doi: 10.1038/s41929-024-01117-4 (PMC11136654; doi:10.1038/s41929-024-01117-4)
Supplement: Supplementary file 1 — Supplementary Figs. 1–16 and Tables 1–8. [file 41929_2024_1117_MOESM1_ESM.pdf]

nature catalysis

Article

https://doi.org/10.1038/s41929-024-01117-4

Epistasis arises from shifting the rate-limiting step during enzyme evolution of a β-lactamase

In the format provided by the authors and unedited

Table of Content

Supplementary Tables 2

Supplementary Table S1: IC_{50} and MIC values against ceftazidime for variants selected during directed evolution. 2

Supplementary Table S2: Overview of IC_{50} and MIC values for all mutational combinations of Q4. 2

Supplementary Table S3: Test for significant changes in IC_{50} for single mutants. 3

Supplementary Table S4: Melting temperatures (T_M) of OXA-48 variants. 3

Supplementary Table S5: Steady state kinetics determined at 25ºC. 3

Supplementary Table S6: X-ray data collection and standard refinement statistics. 4

Supplementary Table S7: Primers used in this study. 5

Supplementary Table S8. List of strains used and constructed in this study. 6

Supplementary Figures 7

Supplementary Figure 1: Epistasis across the fitness landscape. 7

Supplementary Figure 2: Turnover causes partial enzyme inactivation 8

Supplementary Figure 3: Burst-phase kinetics. 9

Supplementary Figure 4: Steady-state Michaelis-Menten kinetics. 10

Supplementary Figure 5: Correlation of *in vitro* k_{cat}/K_M values with *in vivo* IC_{50} values. 11

Supplementary Figure 6: Sequential mixing (burst recovery), size exclusion chromatography and DLS. 12

Supplementary Figure 7: X-ray structure determination. 13

Supplementary Figure 8: Effect of F72L on the Ω-loop conformation within the crystal structures. 15

Supplementary Figure 9: MD simulations of apo wtOXA-48 and Q4. 16

Supplementary Figure 10: MD simulations of the CAZ-acylated OXA-48 variants. 17

Supplementary Figure 11: Changes in per-residue $C_α$ RMSF values compared to wtOXA-48. 18

Supplementary Figure 12: Principal component analysis (PCA) of the OXA-48 variants. 19

Supplementary Figure 13: Cluster analysis of the OXA-48 variants. 20

Supplementary Figure 14: Geometry within the active site. 21

Supplementary Figure 15: Dynamical correlations in the OXA-48 variants. 22

Supplementary Figure 16: Ceftazidime orientation in different β-lactamases. 23

Supplementary References 24

Supplementary Tables

Supplementary Table S1: IC_{50} and MIC values against ceftazidime for variants selected during directed evolution.

Strain no.[a]	Round of evolution	Ceftazidime selection concentration (mg/L)	Mutational background	Ceftazidime IC_{50} (mg/L)	Ceftazidime MIC (mg/L)
MP21-5	-	-	-	0.012 ± 0.002	0.03
MP21-1	-	-	wild-type OXA-48	0.013 ± 0.002	0.03
MP24-51	Round 1	0.25	F72L	0.029 ± 0.006	0.12
MP24-52	Round 2	2	F72L/S212A	0.140 ± 0.003	0.5
MP24-53	Round 3	2	F72L/S212A/T213A (Q3)	0.389 ± 0.025	1
MP24-54	Round 4	8	A33V/F72L/S212A/T213A (Q4)	0.513 ± 0.093	1
MP24-55	Round 5	4	A33V/K51E/F72L/S212A/T213A (Q5)	0.556 ± 0.020	1

[a] see Supplementary Table S8 for a full description of all strains used in this work

Errors are reported as the standard error of the mean based on two replicates.

Supplementary Table S2: Overview of IC_{50} and MIC values for all mutational combinations of Q4.

Strain no.[a]	Amino acids[b]	Number of mutations[c]	Ceftazidime IC_{50} (mg/L)	Ceftazidime MIC (mg/L)
MP21-5	-	-	0.012 ± 0.002	0.03
MP21-1	AFST	0	0.013 ± 0.002	0.03
MP22-7	AFSA	1	0.012 ± 0.001	0.03
MP22-6	AFAT	1	0.015 ± 0.002	0.03
MP22-8	AFAA	2	0.023 ± 0.005	0.06
MP22-5	ALST	1	0.029 ± 0.006	0.12
MP22-38	ALSA	2	0.177 ± 0.063	1
MP22-37	ALAT	2	0.140 ± 0.003	0.5
MP22-39	ALAA (Q3)	3	0.389 ± 0.025	1
MP22-20	VFST	1	0.017 ± 0.004	0.03
MP22-42	VFSA	2	0.015 ± 0.001	0.03
MP22-41	VFAT	2	0.014 ± 0.001	0.03
MP12-47	VFAA	3	0.020 ± 0.003	0.06
MP22-40	VLST	2	0.034 ± 0.002	0.12
MP12-46	VLSA	3	0.141 ± 0.028	0.5
MP12-45	VLAT	3	0.148 ± 0.042	0.5
MP12-48	VLAA (Q4)	4	0.513 ± 0.093	1

[a] see Supplementary Table S8 for a full description of all strains used in this work.

[b] amino acid code referring to the A33V, F72L, S212A and T213A

[c] compared to wtOXA-48.

Errors are reported as the standard error of the mean based on at least two replicates.

Supplementary Table S3: Test for significant changes in IC_{50} for single mutants.

Test details[a]	Adjusted P value
wtOXA-48 vs. A33V	0.7048
wtOXA-48 vs. F72L	0.0060
wtOXA-48 vs. S212A	0.1113
wtOXA-48 vs. T213A	0.9815

[a] Brown-Forsythe one way ANOVA (df=4, $P< 0.001$) followed by Dunnett post hoc test with wtOXA-48 as a control group

Supplementary Table S4: Melting temperatures (T_M) of OXA-48 variants.

OXA-48 variants	Melting temperatures (°C)
wild-type	52.9 ± 0.1
A33V	52.7 ± 0.1
F72L	45.7 ± 0.1
S212A	52.5 ± 0.1
T213A	53.3 ± 0.1
F72L/S212A	45.8 ± 0.1
F72L/T213A	45.6 ± 0.1
F72L/S212A/T213A (Q3)	45.0 ± 0.1
A33V/F72L/S212A/T213A (Q4)	45.3 ± 0.1
A33V/K51E/F72L/S212A/T213A (Q5)	44.2 ± 0.1

Errors are reported as the standard error of the mean based on at three replicates.

Supplementary Table S5: Steady state kinetics determined at 25ºC.

	k_{cat} (s^{-1})	K_M (µM)	k_{cat} / K_M (M^{-1} s^{-1})
wtOXA-48	0.0028 ± 0.0008	247 ± 140	11
F72L	0.0049 ± 0.0004	18 ± 5	281
S212A	NC[a]	NC[a]	24
T213A	NC[a]	NC[a]	31
F72L/S212A	0.0037 ± 0.0003	7 ± 3	565
F72L/T213A	0.0180 ± 0.0005	53 ± 5	339
S212A/T213A	NC[a]	NC[a]	98
Q3	0.0068 ± 0.0003	2 ± 1	3222
Q4	0.0101 ± 0.0004	7 ± 2	1555

[a] NC: Not calculatable due to linearity of the Michaelis-Menten plot.

Errors are reported as the standard error of the mean based on two replicates.

Supplementary Table S6: X-ray data collection and standard refinement statistics.

	F72L	Q5	Q5-CAZ
PBP	8PEA	8PEB	8PEC
Beamline	ID23-EH2, ESRF	ID30B, ESRF	ID30B, ESRF
Wavelength (Å)	0.8731	0.9763	0.9763
Resolution range (Å)	41.41-1.97 (2.04-1.97)	24.95-1.17 (1.19-1.17)	24.28-2.66 (2.76-2.66)
Space group	$P2_12_12_1$	C2	$P6_1$
Unit cell: a,b,c (Å)	64.68, 82.82, 100.99	94.40, 42.54, 64.30	202.15, 202.15, 55.70
α, β, γ (°)	90, 90, 90	90, 106.88, 90	90, 90, 120
Total reflections	277469 (24690)	356104 (16085)	230393 (28723)
Unique reflections	38991 (3822)	82172 (4063)	37116 (3392)
Multiplicity	7.1 (6.5)	4.3 (4.0)	6.1 (6.3)
Completeness (%)	99.74 (99.74)	99.7 (99.9)	99.2 (100.0)
Mean I/sigma(I)	10.05 (0.99)	8.7 (1.1)	9.1 (1.0)
Overall *B*-factor from Wilson plot (Å^2)	37.28	12.7	73.12
R_{merge}	0.1072 (1.352)	0.067 (0.798)	0.108 (1.555)
$R_{measured}$	0.1159 (1.474)	0.086 (1.244)	0.129 (1.846)
R_{pim}	0.04355 (0.5769)	0.041 (0.615)	0.052 (0.761)
$CC_{1/2}$	0.998 (0.675)	0.996 (0.521)	0.997 (0.367)
Resolution range (Å)	41.41-1.97	24.95-1.17	24.39-2.66
Reflections used in refinement	38958 (3818)	82157 (8180)	37109 (3390)
Reflections used for R-free	1613 (159)	1248 (113)	1809 (181)
Final R_{work}	0.2045 (0.3786)	0.1647 (0.3070)	0.2026 (0.3257)
Final R_{free}	0.2474 (0.3992)	0.1871 (0.2873)	0.2713 (0.3631)
No. of non-hydrogen atoms	4264	2557	7669
-macromolecules	3968	2183	7472
-ligands	1	22	113
-solvent	295	352	84
R.m.s. deviations			
-bonds (Å)	0.009	0.009	0.009
-angles (°)	1.19	1.16	1.09
Ramachandran plot			
-Favoured (%)	94.58	98.75	92.597
-Allowed (%)	3.33	1.25	6.58
-Outliers (%)	0.95	0.00	0.45
Average B-factor (Å^2)	45.74	23.67	95.81
-macromolecules (Å^2)	45.69	21.77	96.04
-ligands (Å^2)	35.79	27.91	93.80
-solvent (Å^2)	46.41	35.18	78.18

Statistics for the highest-resolution shell are shown in parentheses.

Supplementary Table S7: Primers used in this study.

No.	Name		5´-3´	Ref.
3	pUN-NcoI	F	GCTTTCCCATGGATGTTTTTCCTCCTTATGTTAAGCTTACTCAG	[1]
4	pUN-XhoI	F	GCTTCTCGAGAAGTGGTTAGCGCGTATTTGTG	[1]
7	preOXAseq	F	GATTACGCGCAGACCAAAACG	[1]
8	postOXAseq	R	CCTATTTCCCTAAAGGGTTTATTGAGAATATG	[1]
15	F72L	F	TTTTTTGCTCTTCGCATCTACCCTGAAAATTCCCAATAGCTT	[1]
		R	TTTTTTGCTCTTCGATGCGGGTAAAAATGCTTG	
16	S212A	F	TTTTTTGCTCTTCACTGGATACGCGACTAGAATCGAACCTAAGATTGG	[1]
		R	TTTTTTGCTCTTCCCAGTTTTAGCCCGAATAATATAGTCACC	
17	T213A	F	TTTTTTGCTCTTCACTGGATACTCGGCGAGAATCGAACCTAAGATTGG	[1]
		R	P16R	
18	S212A/T213A	F	TTTTTTGCTCTTCACTGGATACGCGGCGAGAATCGAACCTAAGATTGG	This study
		R	P16R	
25	A33V	F	TTTTTTGCTCTTCAGTTGGAATGTTCACTTTACTGAACAT	This study
		R	TTTTTTGCTCTTCCAACTTTTGTTTTCTTGCCATTC	
35	pDest17 vector-NotI	F	TTTTTTGCGGCCGCTTCGAGGTGATGGTGATGGTGATGGTAGTACGACATA	This study
36	pDest17 vector-XhoI	R	TTTTTTCTCGAGTGATTCGAGGCTGCTAACAAAGCCCG	This study
37	NotI-TEV-OXA-48	F	TTTTTTGCGGCCGCAGAGAACCTGTATTTTCAGGGTAAGGAATGGCAAGAAAACAAAAGTTGG	This study
		R	P2R	

F: forward primer, R: reverse primer

Supplementary Table S8. List of strains used and constructed in this study.

Strain no.	Strain name [a]	Vector number	Insert	Reference
21-5	E. cloni	None	None	Lucigen
21-1	E. cloni	pUNe-4	wtOXA-48	[1]
Clones selected after error prone PCR:				
21-9	E. cloni	pUNe-5	F72L	This study
21-21	E. cloni	pUNe-8	F72L/S212A	This study
21-30	E. cloni	pUNe-11	F72L/S212A/T213A	This study
21-62	E. cloni	pUNe-14	A33V/F72L/S212A/T213A	This study
21-73	E. cloni	pUNe-17	A33V/K51E/F72L/S212A/T213A	This study
Subcloned into isogenic vector and strain backbone after selection:				
24-51	E. cloni	pUNe-5.1	F72L	This study
24-52	E. cloni	pUNe-8.1	F72L/S212A	This study
24-53	E. cloni	pUNe-11.1	F72L/S212A/T213A	This study
24-54	E. cloni	pUNe-14.1	A33V/F72L/S212A/T213A	This study
24-55	E. cloni	pUNe-17.1	A33V/K51E/F72L/S212A/T213A	This study
Mutants constructed for the landscape:				
22-5	E. cloni	pUNs-1	F72L	[1]
22-6	E. cloni	pUNs-2	S212A	[1]
22-7	E. cloni	pUNs-3	T213A	[1]
22-8	E. cloni	pUNs-4	S212A/T213A	This study
22-20	E. cloni	pUNs-17	A33V	This study
22-37	E. cloni	pUNs-34	F72L/S212A	[1]
22-38	E. cloni	pUNs-35	F72L/T213A	This study
22-39	E. cloni	pUNs-36	F72L/S212A/T213A	This study
22-40	E. cloni	pUNs-37	A33V/F72L	This study
22-41	E. cloni	pUNs-38	A33V/S212A	This study
22-42	E. cloni	pUNs-39	A33V/T213A	This study
12-45	E. cloni	pUNs-121	A33V/F72L/S212A	This study
12-46	E. cloni	pUNs-122	A33V/F72L/T213A	This study
12-47	E. cloni	pUNs-123	A33V/S212A/T213A	This study
12-48	E. cloni	pUNs-124	A33V/F72L/S212A/T213A	This study
Mutants constructed for protein expression (without signal peptide):				
12-71	E. cloni	pURR-1	wtOXA-48	This study
12-72	E. cloni	pURR-2	F72L	This study
12-73	E. cloni	pURR-3	S212A	This study
12-74	E. cloni	pURR-4	T213A	This study
12-75	E. cloni	pURR-5	F72L/S212A	This study
12-76	E. cloni	pURR-6	F72L/T213A	This study
12-77	E. cloni	pURR-7	F72L//S212A/T213A	This study
24-16	E. cloni	pURR-14	A33V/F72L/S212A/T213A	This study
13-78	E. cloni	pDEST-17	A33V/K51E/F72L/S212A/T213A	This study
Strains for protein expression:				
13-02	BL21 AI	None	None	ThermoFisher
24-01	BL21 AI	pURR1	wtOXA-48	This study
24-02	BL21 AI	pURR-2	F72L	This study
24-03	BL21 AI	pURR-3	S212A	This study
24-04	BL21 AI	pURR-4	T213A	This study
24-05	BL21 AI	pURR-5	F72L/S212A	This study
24-06	BL21 AI	pURR-6	F72L/T213A	This study
24-07	BL21 AI	pURR-7	F72L//S212A/T213A	This study
24-08	BL21 AI	pURR-14	A33V/F72L/S212A/T213A	This study
13-80	BL21 AI	pURR-11	A33V/K51E/F72L/S212A/T213A	This study

[a] Species for all strains is *E. coli.*

Supplementary Figures

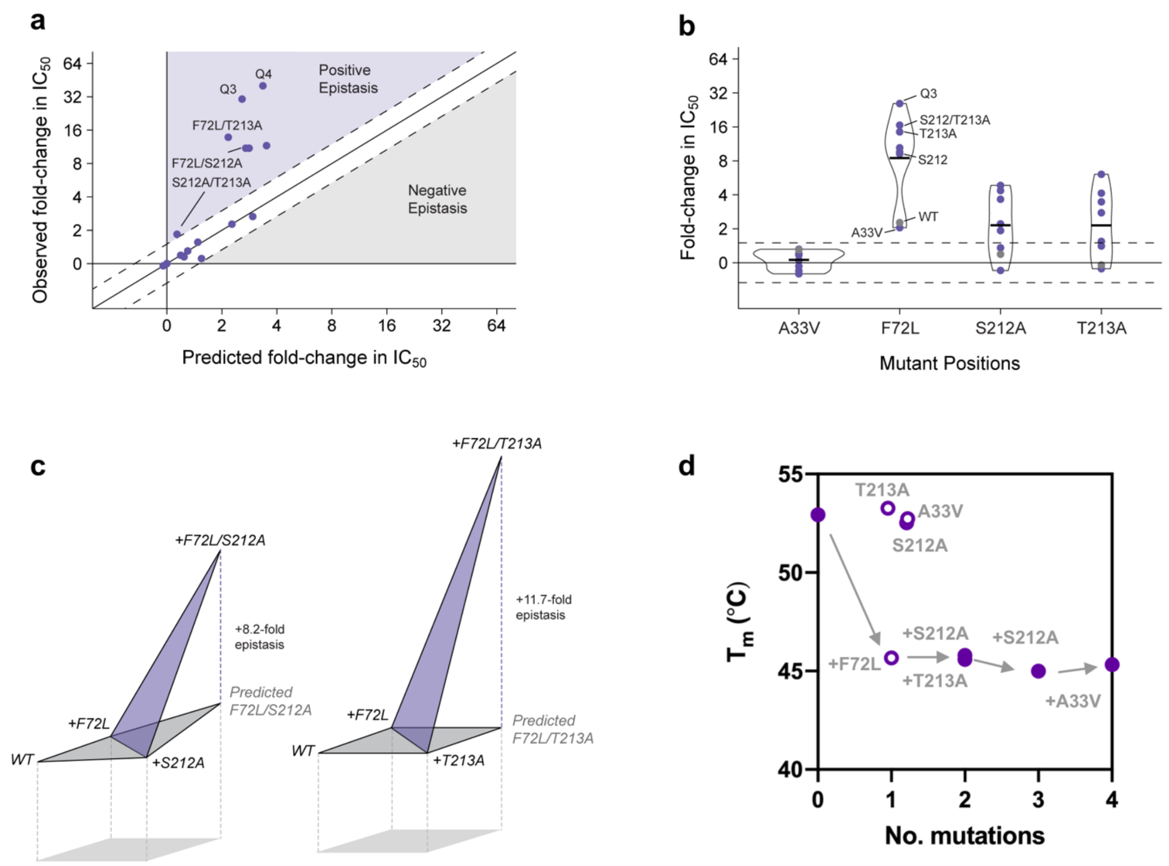

Supplementary Figure 1: Epistasis across the fitness landscape.

a. Predicted IC_{50} fold changes based on additive contributions of single mutational effects in wtOXA-48. Genotypes were positively epistatic (purple) when observed fold-changes exceeded additive predictions by more than 1.5-fold and negatively epistatic (grey) when less than 1.5-fold. **b.** Contribution of mutations on the IC_{50} fold-change in each genotypic background. Distributions are shown by violin plots with bars representing mean fold-change in IC_{50} and grey points representing the wtOXA-48 background. The dashed lines in **a** and **b** represent arbiturarily defined error bands (1.5-fold). **c.** Positive epistasis is particularly apparent in the combinations of F72L with either S212A or T213A. **d.** Changes in melting temperature (T_m) for single and multistep mutants during the directed evolution. Open circles represent single mutants, and filled circles mutational combinations.

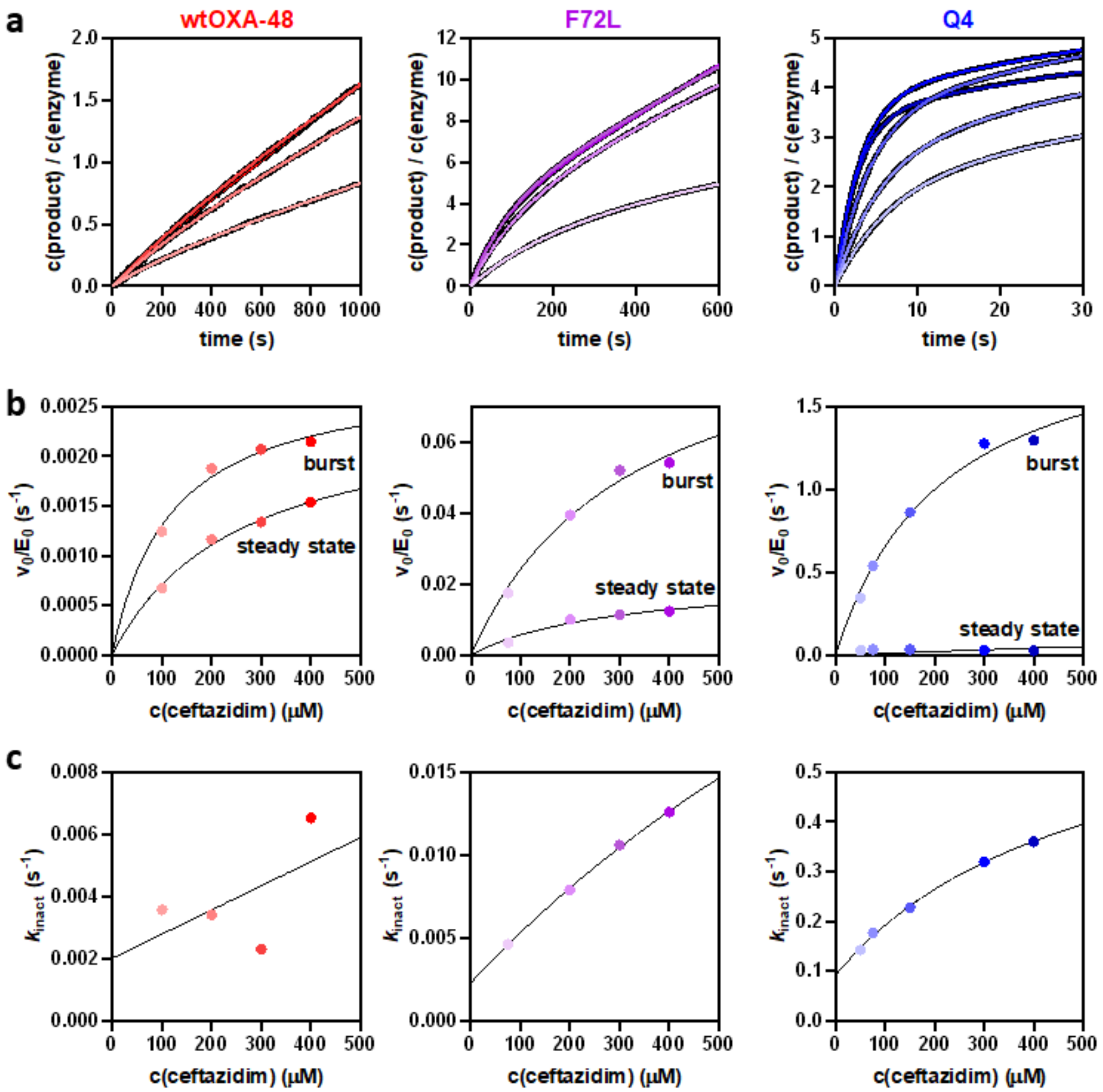

Supplementary Figure 2: Turnover causes partial enzyme inactivation

a. Turnover of CAZ by wtOXA-48 (red), F72L (purple) and Q4 (blue) partially inactivates the enzymes over several turnovers (light to dark colors indicate increasing CAZ concentrations). **b.** and **c.** Fitting of the curves in **a.** to Eq. 1 allows extracting the burst and steady-state activities (v_0/E_0, **b**) and inactivation rates (k_{inact}, **c**). The activity difference between burst and steady state increased during evolution, indicating selective improvement of the burst-phase ensemble. k_{inact} was fitted to a Michaelis-Menten type-model with an added constant c ($k_{inact} = k_{inact,max} * c(CAZ) / (K_M^* + c(CAZ) + c)$. k_{inact} rises with increasing CAZ concentrations, supporting that substrate binding induces inactivation.[2] Note that K_M^* may not be similar to K_M.

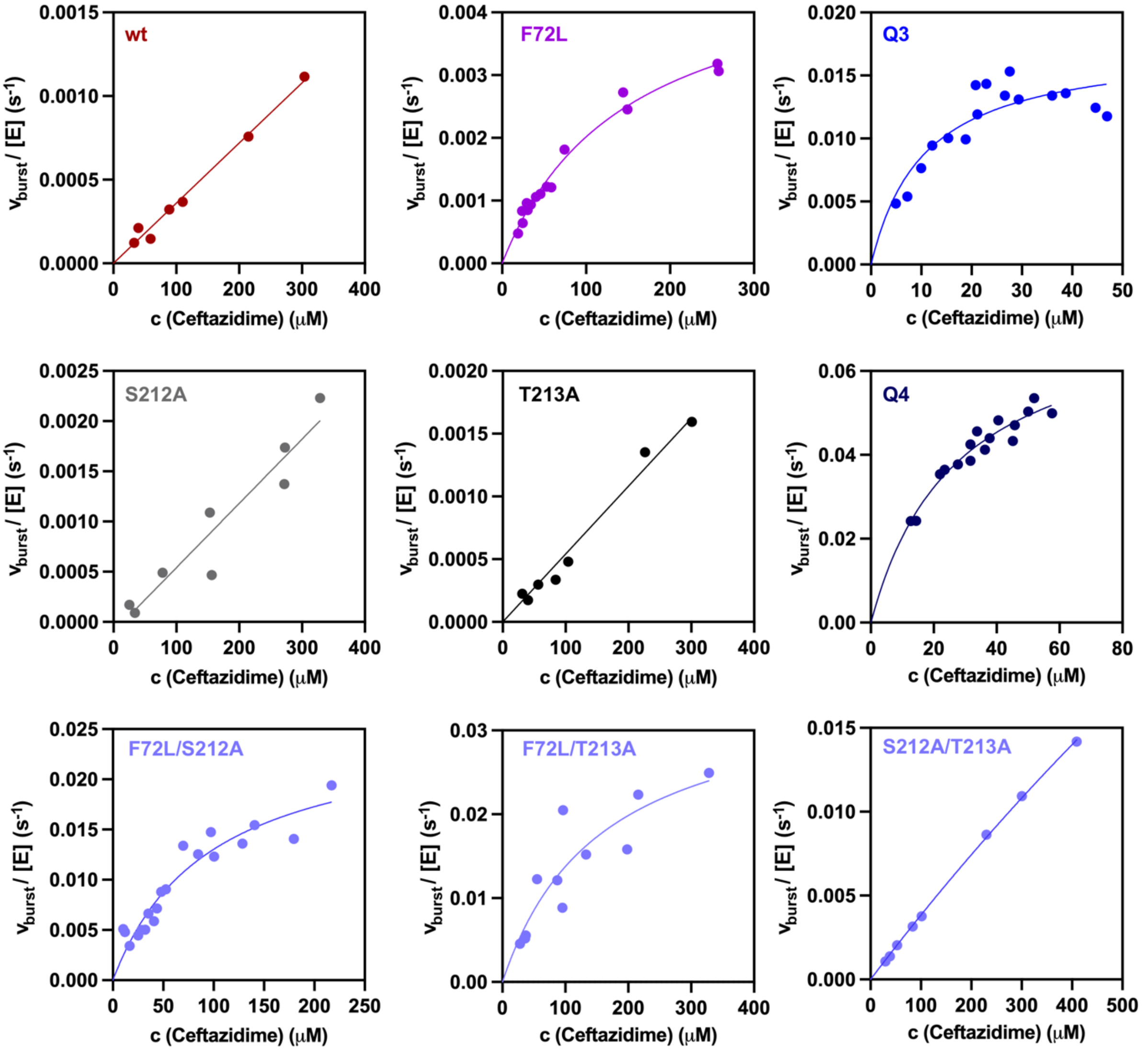

Supplementary Figure 3: Burst-phase kinetics.

The burst-phase kinetics reveal that F72L predominantly improves CAZ affinity (approximated by K_M), while S212A and T213A improve catalysis (k_{cat}). The *in vitro* burst-phase k_{cat}/K_M values correlate well with the *in vivo* IC_{50} values (Supplementary Figure 4). Burst-phase activities were determined at 4°C.

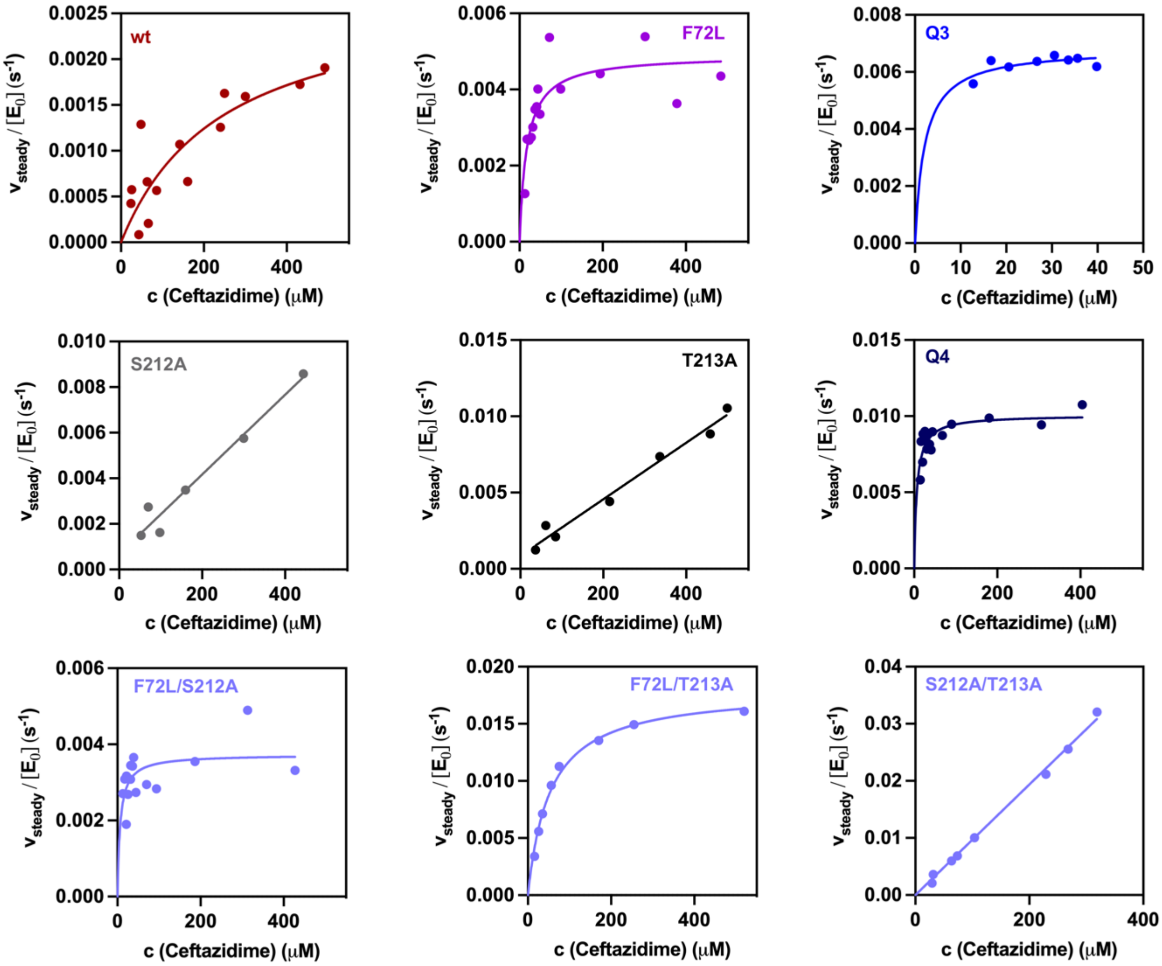

Supplementary Figure 4: Steady-state Michaelis-Menten kinetics.

The Michaelis-Menten kinetics of the steady-state for various OXA-48 variants reveal similar overall trends compared to our burst phase results (Supplementary Figure S3). F72L predominantly improves CAZ affinity (approximated by K_M), while S212A and T213A improve catalysis (k_{cat}). Nevertheless, the epistasis evident from the IC_{50} values is not as pronounced in the steady-state as in the burst-phase kinetics (Supplementary Figure 3 and 5). Activities were determined at 25ºC.

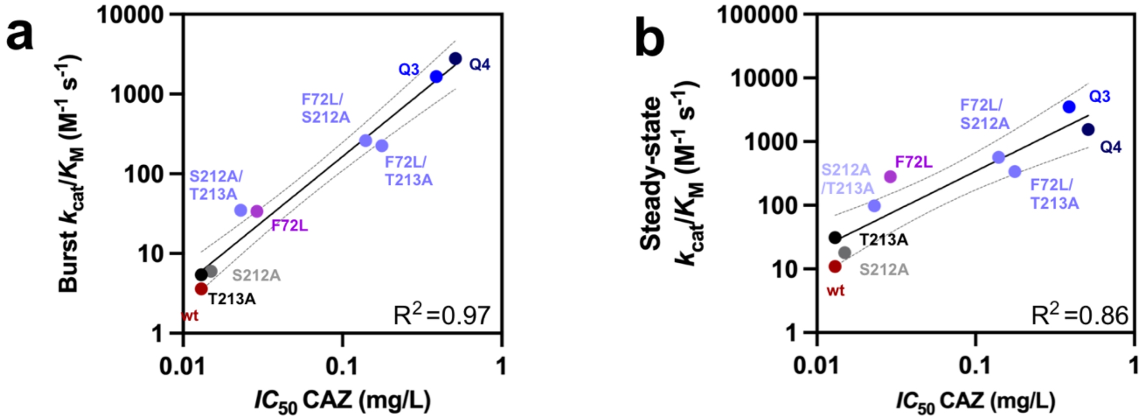

Supplementary Figure 5: Correlation of *in vitro* k_{cat}/K_M values with *in vivo* IC_{50} values.
a. The burst-phases k_{cat}/K_M values correlate mores strongly with IC_{50} (R^2 = 0.97) than **b.** k_{cat}/K_M values obtained from steady-state kinetics (R^2 = 0.86). Burst-phase data were obtained at 4ºC, and steady-state measurements were performed at 25ºC. The line represents the Pearson correlation and the error bands display the 95% confidence interval.

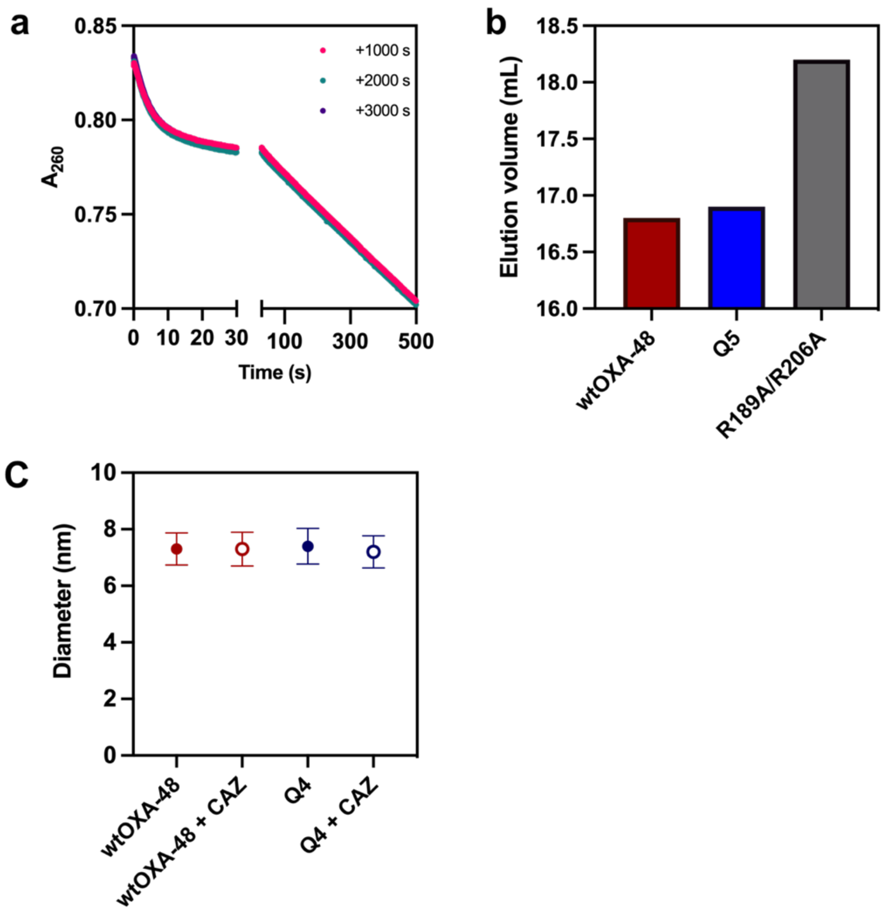

Supplementary Figure 6: Sequential mixing (burst recovery), size exclusion chromatography and DLS.

a. Repeated sequential mixing using Q4 demonstrates the full recovery of the enzyme after each burst. **b.** Elution volume between wtOXA-48, Q5, and the monomeric wtOXA-48 mutant R189A/R206A[3] indicate that Q5 remains dimeric under the assay conditions. **c.** Dynamic light scattering of wtOXA-48 and Q4 before (full circles) and after (empty circles) incubation with 400 µM CAZ. Errors are reported as the standard error of the mean based on nine replicates.

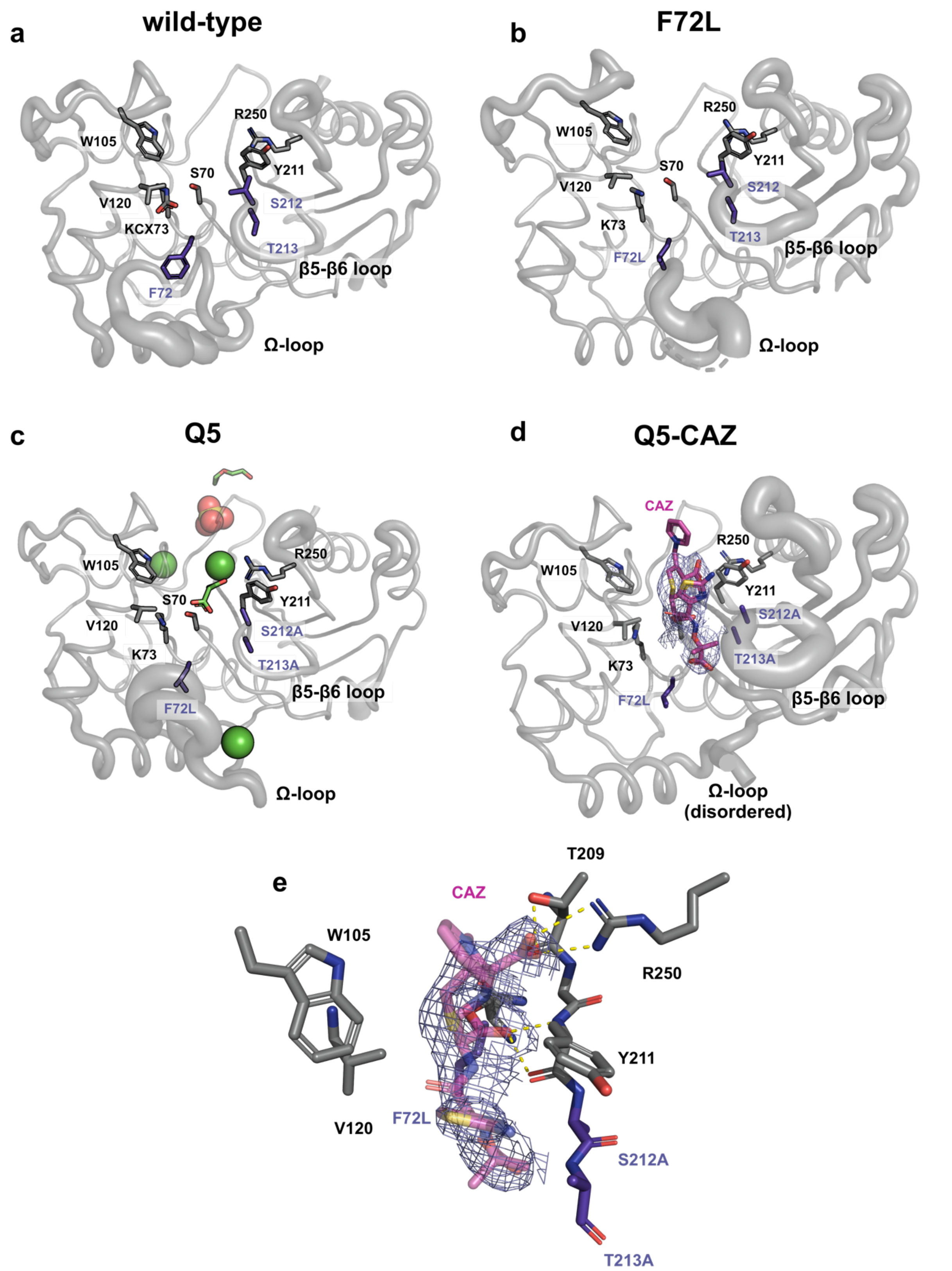

Supplementary Figure 7: X-ray structure determination.

Common active site residues relevant for binding and catalysis (S70, K73, W105, V120 and R250) are shown in grey and mutational spots are colored in purple (F72, S212 and T213) in each average X-ray crystal structure. For general crystallographic information and refinement

statistics see Tab. S6. Electron densities maps are shown as 2Fo-Fc maps at 1σ. **a.** Structure of wtOXA-48 (PDB ID: 4S2P[4], 1.70 Å) is shown for comparison. Lysine K73 is displayed in its carbamylated form (KCX73). **b.** The crystal structures of F72L (PDB ID: 8PEA, chain B shown) was solved to 1.97 Å and with two molecules in the asymmetric unit, forming a dimer with one chlorine ion at the dimer interface, as described previously.[3] The Ω-loop in chain A (residues 148 to 161) and B (residues 149 to 153) displayed poor electron density thus were refined with zero occupancy. **c.** The crystal structure of Q5 was resolved to 1.17 Å (PDB ID: 8PEB) with one molecule in the asymmetric unit and the final model include three chlorine (green spheres), one sulfate and two PEG molecules (green sticks). A dimer of Q5 is obtained through the crystallography symmetry. **d.** Crystals of Q5 were soaked for <5 min in CAZ resulting in the Q5-CAZ structure to 2.66 Å (PDB ID: 8PEC). We identified four protein chains in the asymmetric unit of Q5-CAZ where the chains A/B and C/D each formed a dimer with one chlorine in each dimer interface, as described previously.[3] Chains A, C and D exhibited sufficient electrons density within the active site, allowing the refinement of covalently bound CAZ in these three chains with averaged occupancies of 0.64, 0.55 and 0.63, respectively (figure displays chain A). None of the CAZ pyridine rings were defined in any of the chains, thus given zero occupancy. Similar to previous CAZ bound OXA-48 structure[5], there is no electron density for the residues within the Ω-loop in chain A (147 to 160), chain B (147 to 158), chain C and D (147 to 161). **e.** CAZ within the active site of Q5 was involved in H-bonds (main chains S70, Y211 and side chain of T209) as well as electrostatic interactions with R250.

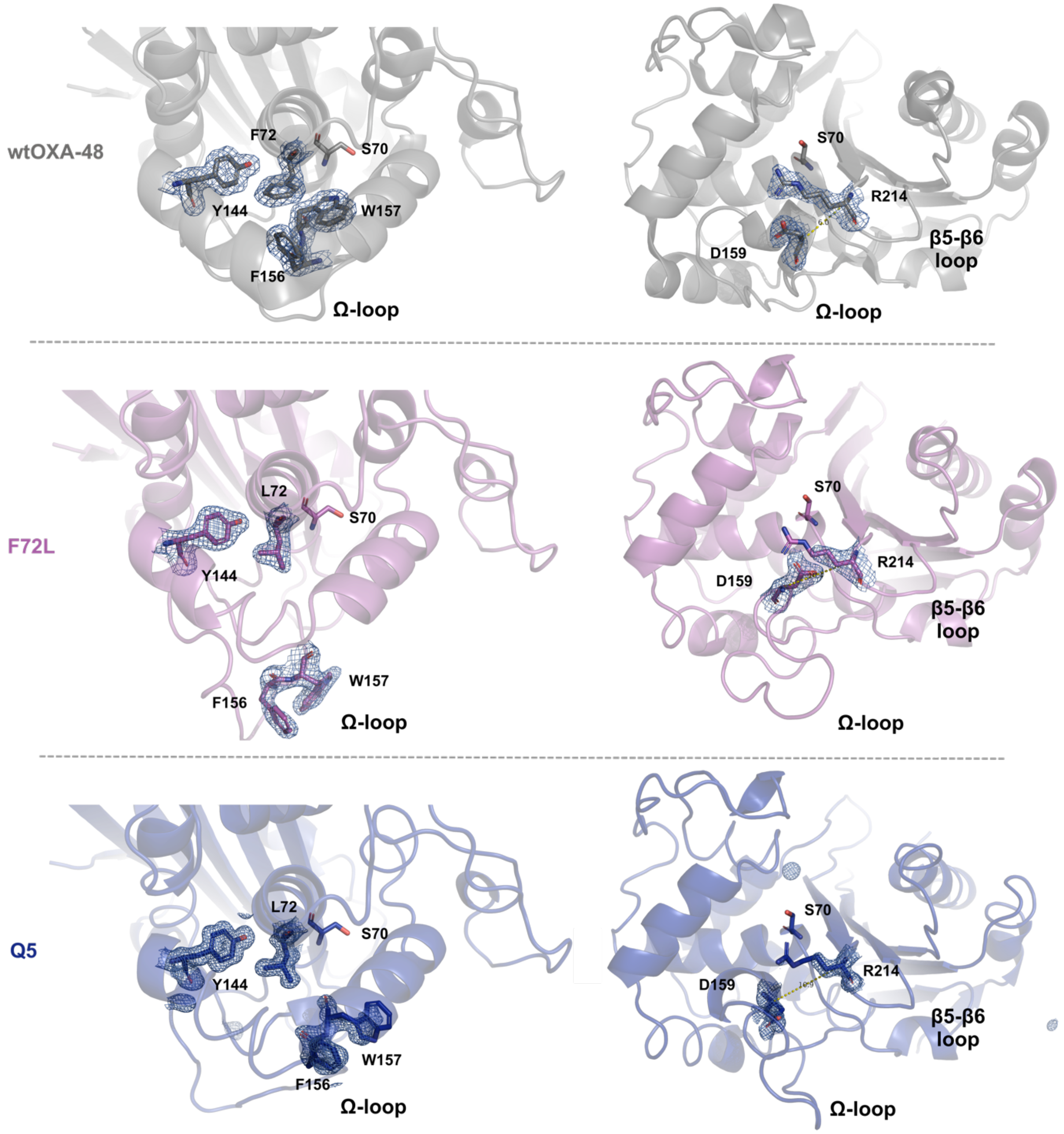

Supplementary Figure 8: Effect of F72L on the Ω-loop conformation within the crystal structures.

In wtOXA-48 (4S2P[4], chain A displayed), F72 is located next to the active site S70 and anchored within an aromatic cluster formed by Y144, F156 and W157 (left). F156 and W157 are located within the Ω-loop and the stability of this loop has been reported[6] to be promoted by the electrostatic interactions between D159 (in the Ω-loop) and R214 within the β5-β6 loop (C_α-C_α distance of 6 Å, right). We find that with the introduction of F72L into the wt structure (8PEA, chain B displayed) the aromatic cluster becomes disturbed, allowing the average structure of the Ω-loop, including F156 and W157, to move out of the active site and adopt a different conformation (Fig. 3a). The perturbation of this aromatic network is also maintained in Q5 (8PEB) and has knock on effect on other parts of the structure, including D159 and R214, demonstrated by the increased C_α-C_α distance (10 Å) and much less refined electron density of R214. Electron density maps are shown as 2Fo-Fc maps at 1σ.

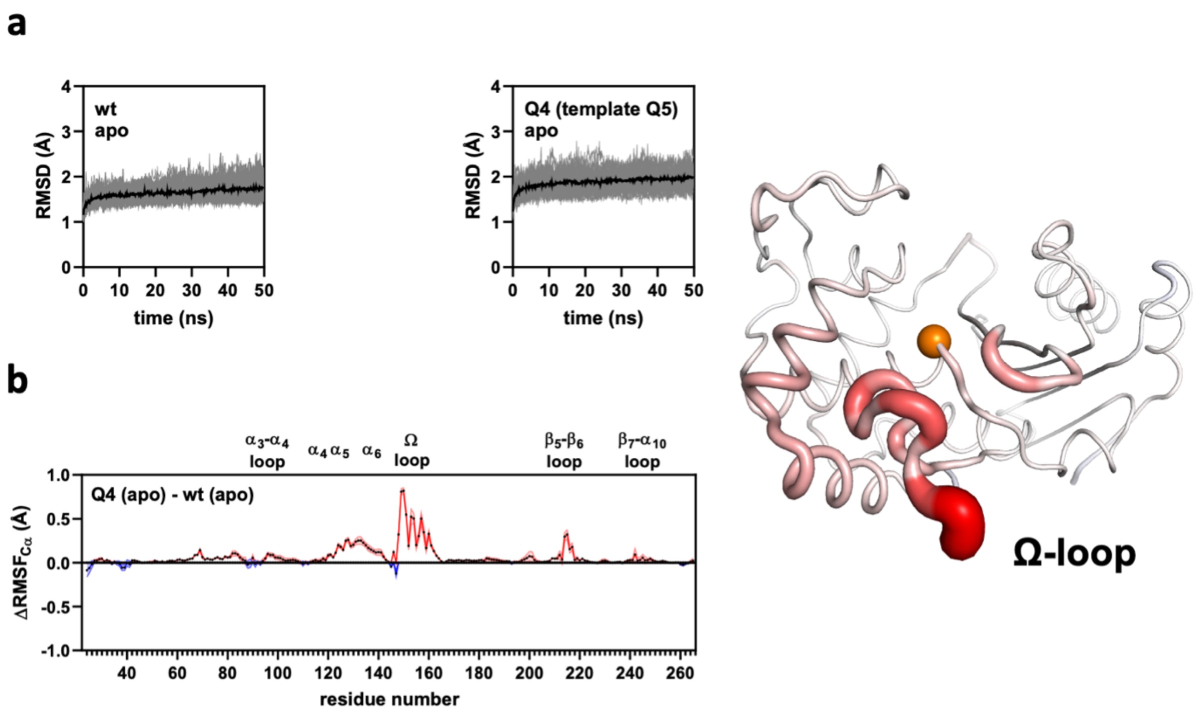

Supplementary Figure 9: MD simulations of apo wtOXA-48 and Q4.

a. RMSD plots of apo wtOXA-48 (based on PDB ID: 4S2P[4]) and apo Q4 (based PDB ID: 8PEB, Q5) indicate that the simulations are stable over 50 ns (black line represents the average of 40 the replicates shown in grey). **b.** apo Q4 is substantially more flexible than wtOXA-48, especially in the Ω-loop region as highlighted in the structure to the right (error bars reflect the standard error of the mean from 40 replicates). Orange sphere indicated the active site S70. The increased Ω-loop flexibility likely aids substrate entry.

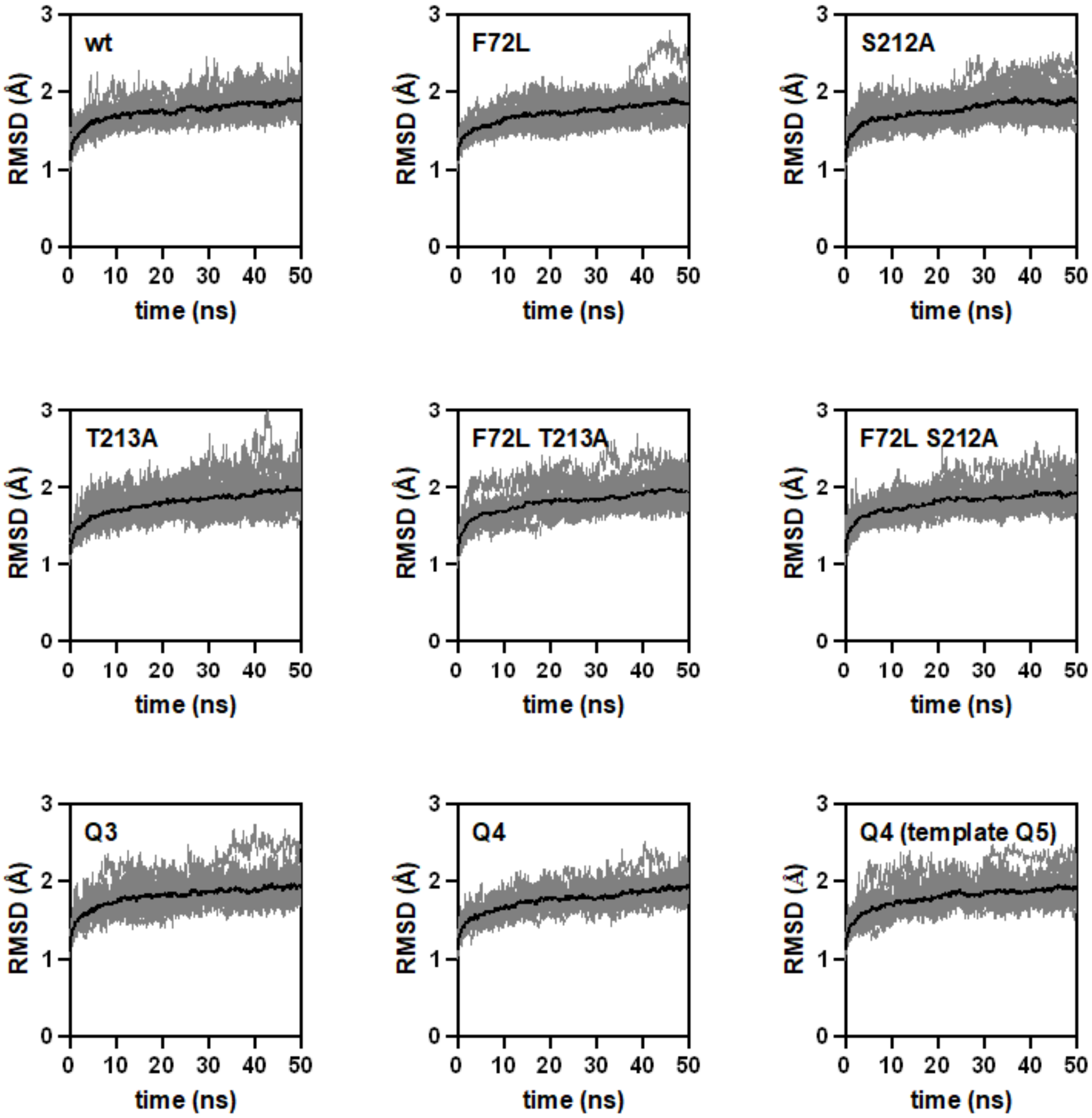

Supplementary Figure 10: MD simulations of the CAZ-acylated OXA-48 variants. RMSD plots for each variant indicate that the simulations are stable over 50 ns. The first 10 ns of each simulation were omitted from the subsequent analysis. The black line reflects the average of 40 independent replicates (grey). RMSD values were calculated based on the C_α coordinates.

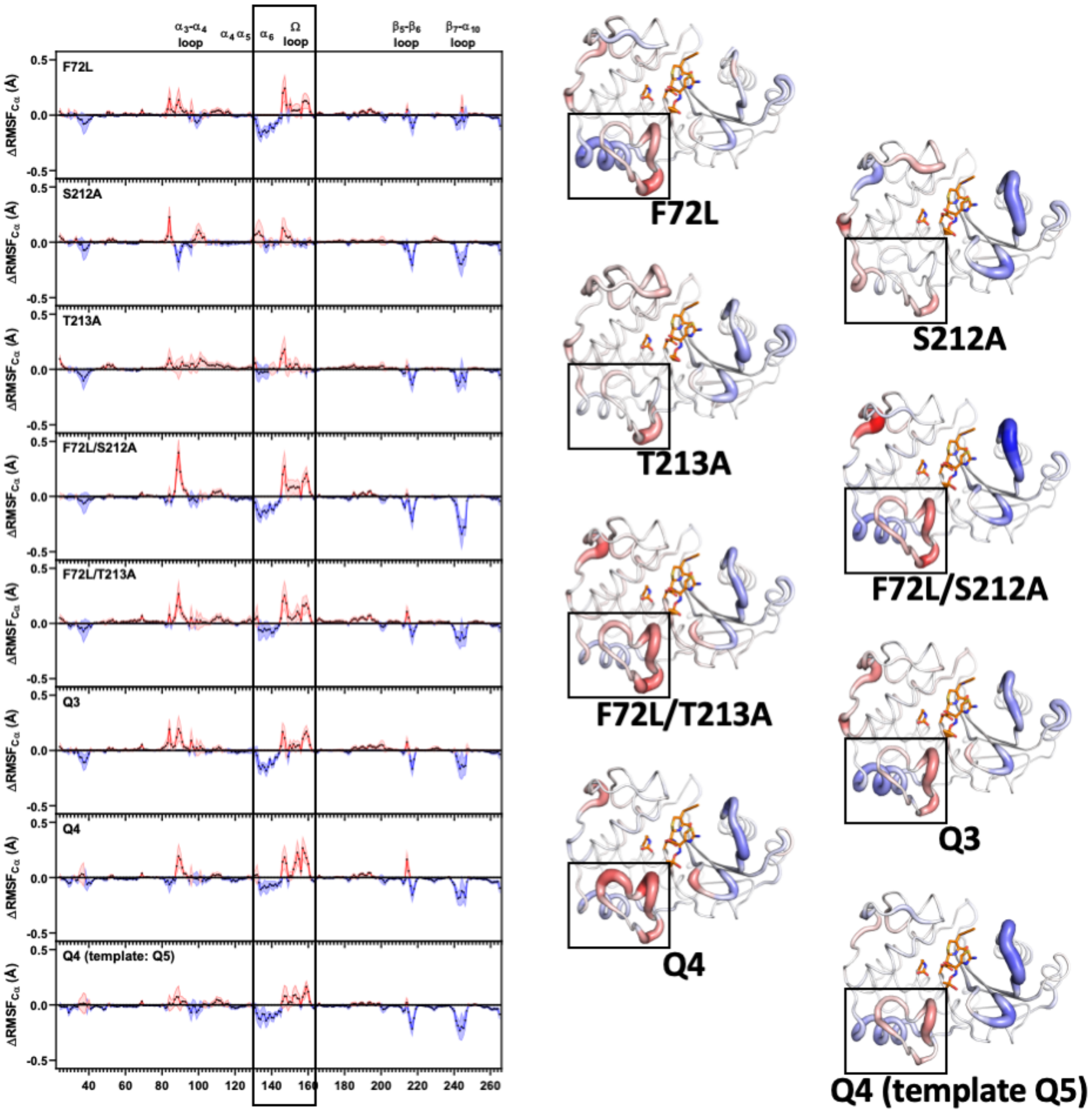

Supplementary Figure 11: Changes in per-residue C_α RMSF values compared to wtOXA-48.

MD simulations reveal that F72L remodels flexibility in OXA-48, particularly in the Ω loop. Residues that become more flexible compared to wtOXA-48 are shown in red, and those that become more rigid are shown in blue. In the structures, the tube thickness and color intensity are scaled to the overall change in dynamics. We note that the RMSF calculations tend to require more MD data to converge compared to the cluster analysis and dynamical correlations (Supplementary Figure 12 to 15) and are thus comparably noisier (error bars reflect the standard error of the mean from 40 replicates). Therefore, the dynamics of the system were subsequently analyzed using principal component (Supplementary Figure 12), cluster (Supplementary Figure 13), and dynamical correlation analysis (Supplementary Figure 15).

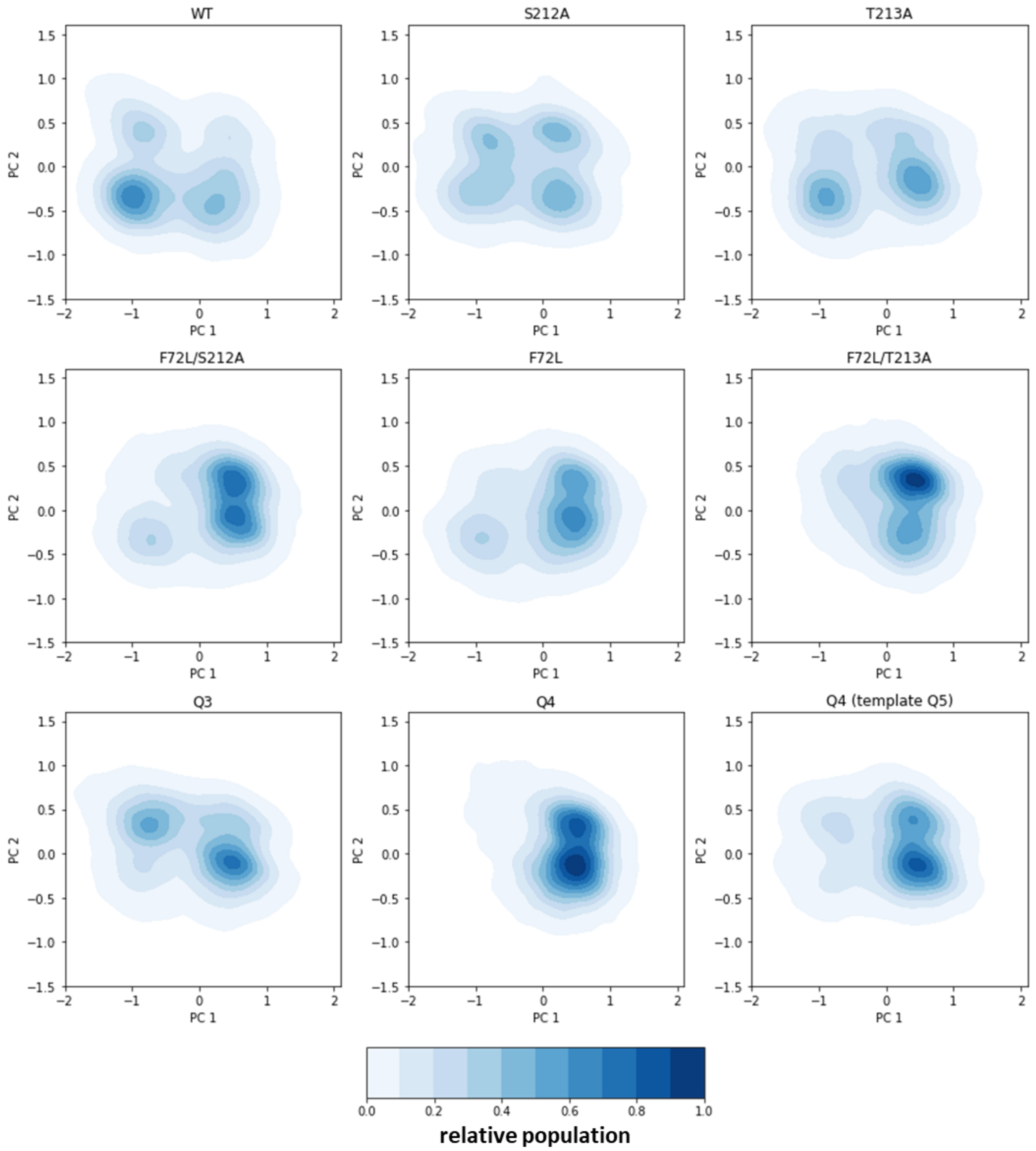

Supplementary Figure 12: Principal component analysis (PCA) of the OXA-48 variants. The F72L mutation changes the explored conformational landscape of OXA-48. The conformational landscape is only changed upon adding F72L, as revealed by PC analysis. Notably, the S212A and T213A mutations only marginally affect the shape of the landscape in any genetic background.

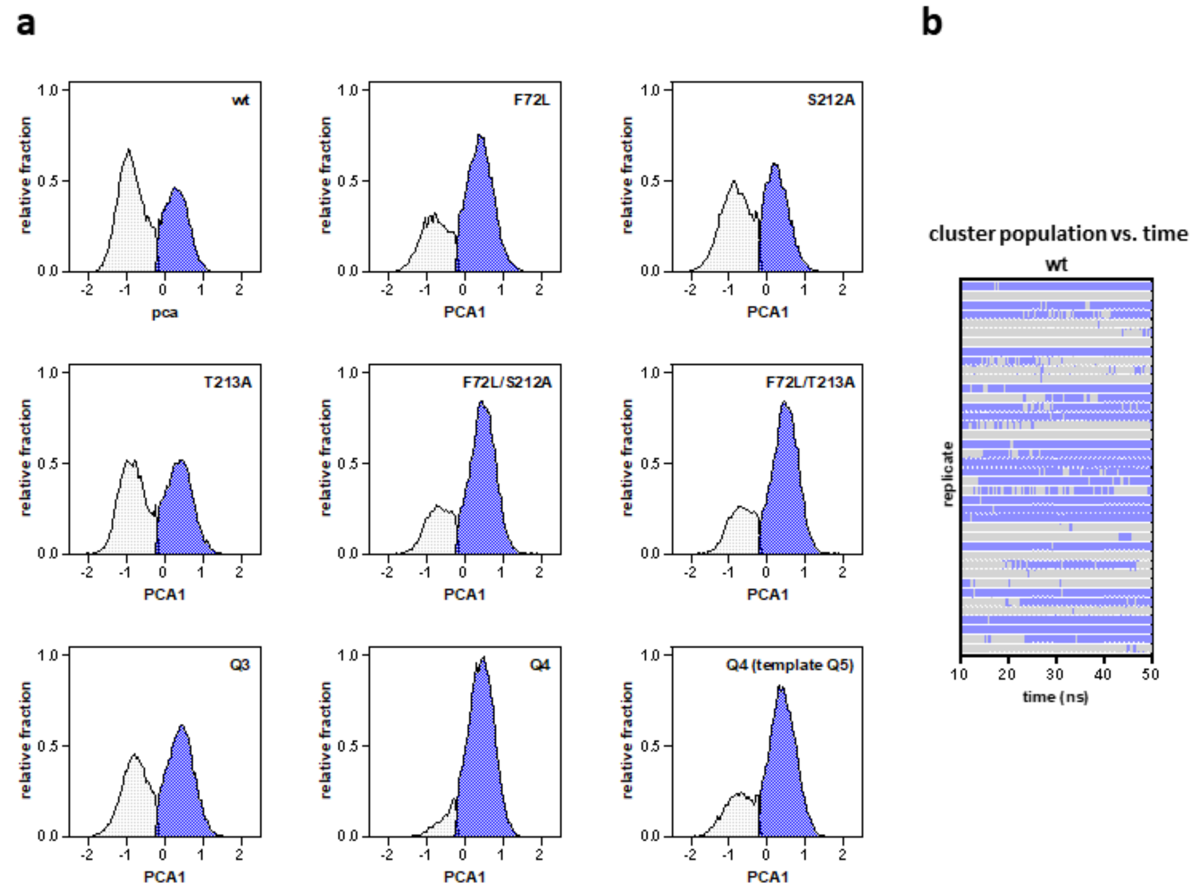

Supplementary Figure 13: Cluster analysis of the OXA-48 variants.

a. Cluster analysis was performed based on C_α RMSD values. F72L modulates the conformational ensemble of the loops, leading to an increase population of a distinct state (blue). The MD trajectories (of all variants) were partitioned into two clusters using the k-means algorithm and projected onto PC1. We note that the PC analysis (Fig. S12) indicates a broader conformational diversity than a two-state cluster model and that the two clusters thus represent a larger group of conformationally-similar sub-states. Nonetheless, a two-state model sufficiently captures the overall perturbations induced by F72L observed in the conformational ensemble. **b.** Analysis of the cluster population against time reveals that most simulations sample multiple transitions between the clusters. Here, the analysis is shown for all 40 replicate simulations of wtOXA-48, but similar observations were made for all other variants (grey: initial conformation, blue: evolved conformation).

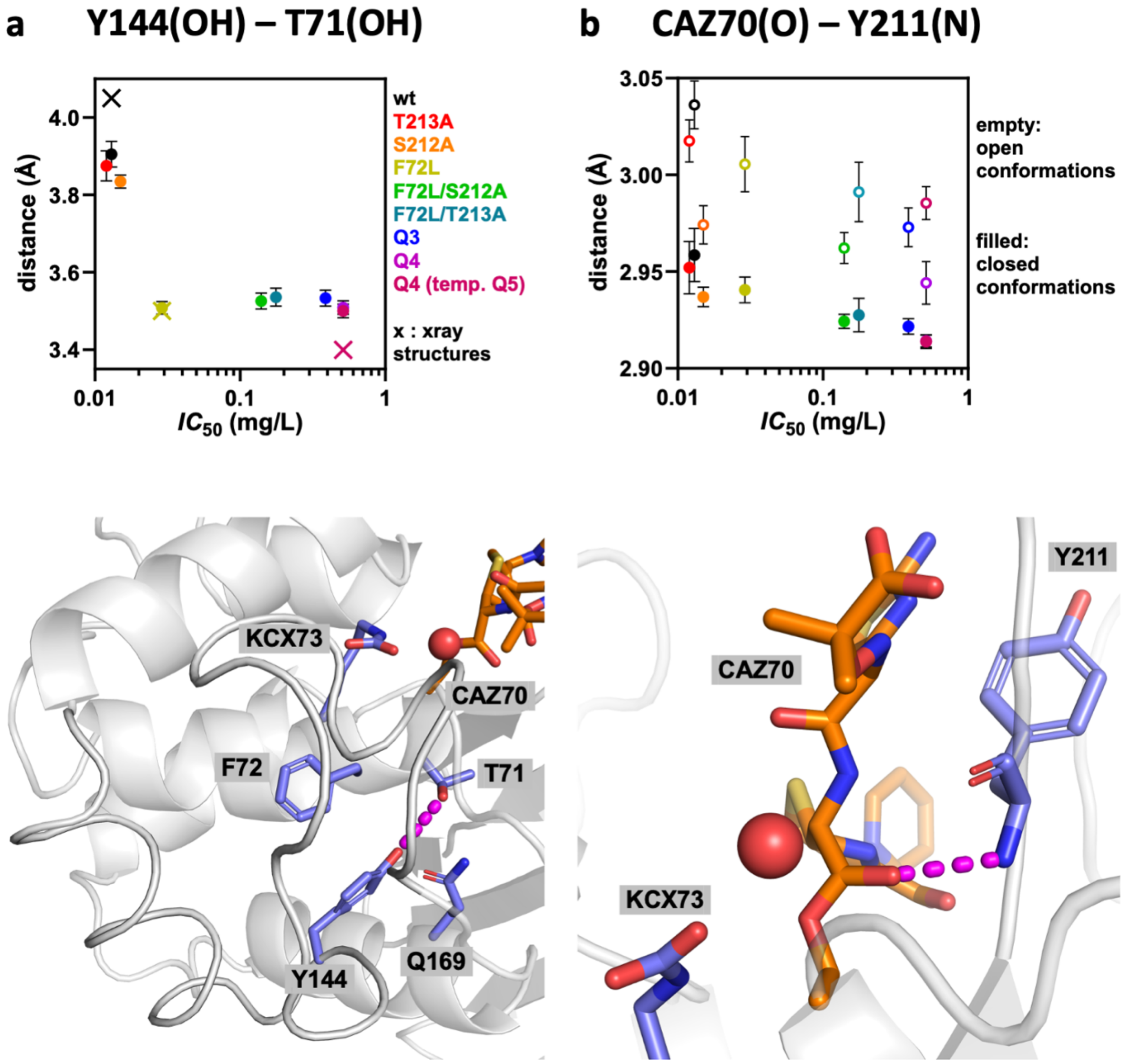

Supplementary Figure 14: Geometry within the active site.

a. The distinct effect of F72L is exemplified by its effect on the H-bonding network comprising T71, Y144, and Q169. The determined distance between Y144 – T71 is highlighted with the purple dashed line on the bottom. The carbamylated K73 is labeled as KCX73 and the CAZ-acylated S70 as CAZ70 (orange). Interestingly, the trends observed for the hydrogen-bonding network during the MD simulations reproduce the distances extracted from the apo structures (indicated as crosses). **b.** The oxyanion hole is formed by the backbone amides of Y211 and S70. The distance between the CAZ70 oxyanion and Y211(N) is successively decreased during evolution, signaling better oxyanion stabilization. Notably, the closed states (filled circles) show significantly shorter interaction than the respective open states (empty circles; CAZ70(O) – Y211(N), distance is highlighted with the purple dashed line). Error bars represent the standard error of the mean for 40 replicates.

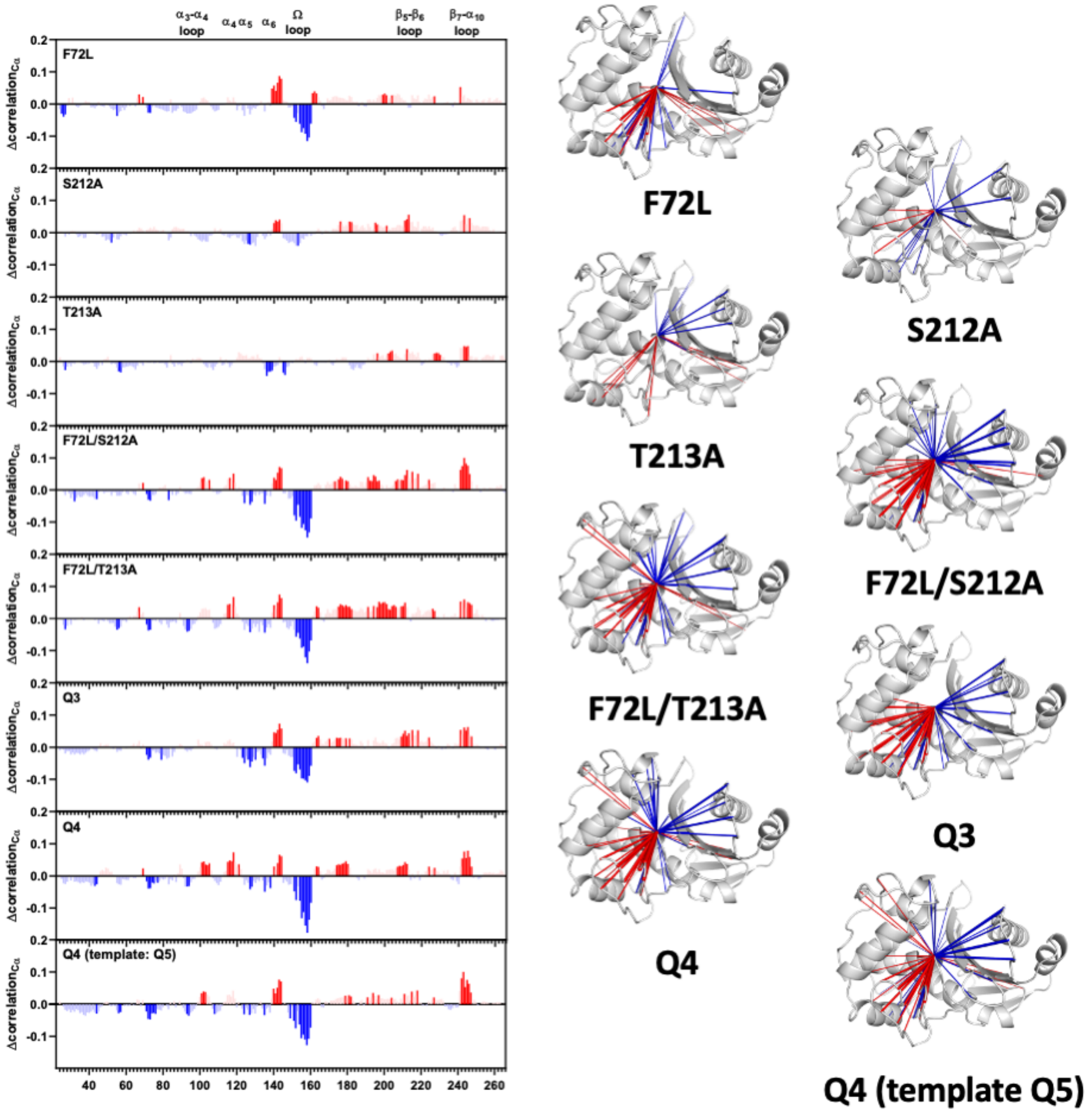

Supplementary Figure 15: Dynamical correlations in the OXA-48 variants.

Changes in dynamical correlations of S70 with other protein residues are shown relative to wtOXA-48. The changes in correlations reveal distinct effects for F72L and the alanine mutations. F72L primarily reduced the correlation of S70 with the Ω-loop. In contrast, the alanine mutations increase correlation, particularly with the oxyanion-hole harboring half of the protein scaffold. Residues that become more correlated with S70 are highlighted in blue, and those that are less correlated in red. Only statistically significant changes compared to wtOXA-48 (t-test, $\alpha = 0.05$, dark colors, 40 replicates per variant) are shown on the structures. The width of the lines in the structures corresponds to the magnitude of change in correlation.

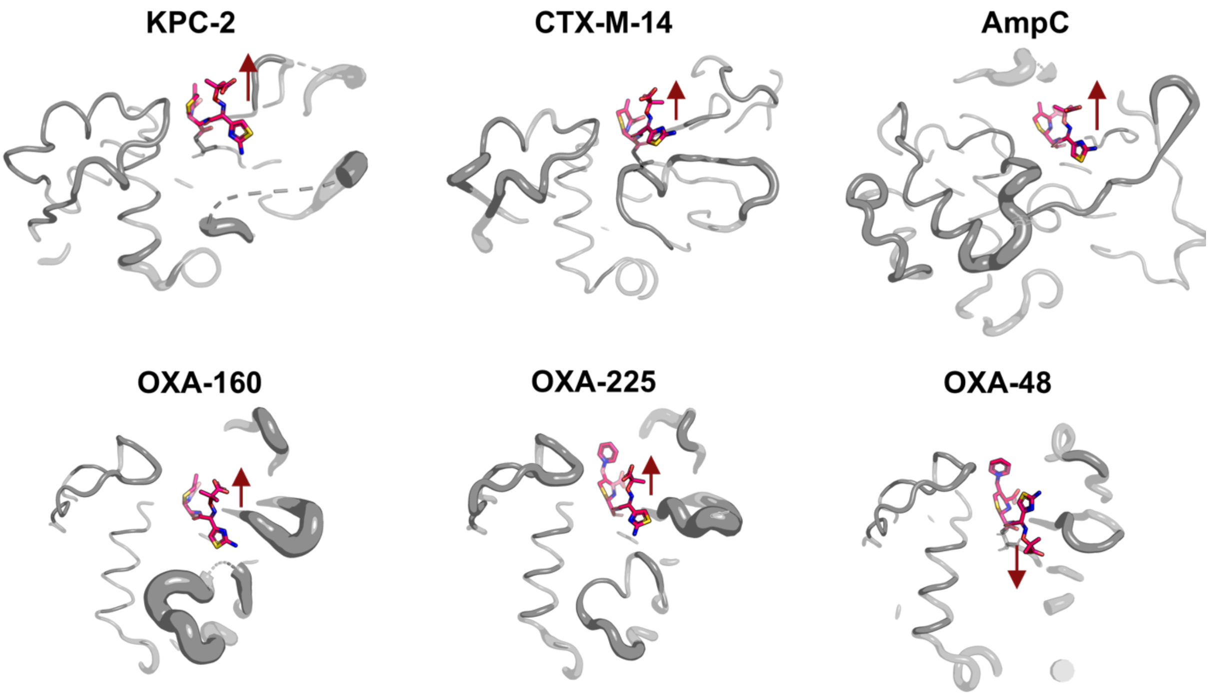

Supplementary Figure 16: Ceftazidime orientation in different β-lactamases.

OXA-48 shows a distinct orientation of ceftazidime in the active site compared to other β-lactamases. Position of ceftazidime (pink) in the active site of KPC-2:E166Q (PDB ID: 6Z24[7]), CTX-M-14:E166A (PDB ID: 5U53[8]), AmpC (PDB ID: 1IEL[9]) as well as OXA-160:V130D (PDB ID: 4X56[10]), OXA-225:K82D (PDB ID: 4X55[10]) and OXA-48:P68A (PDB ID: 6Q5F[5]). The direction of the oxyimino group of ceftazidime is indicated by a red arrow, which points outwards from the active site for all depicted enzymes except for OXA-48:P68A.

Supplementary References

1. Fröhlich, C., Sørum, V., Tokuriki, N., Johnsen, P.J. & Samuelsen, Ø. Evolution of β-lactamase-mediated cefiderocol resistance. *J Antimicrob Chemother*, 2429–2436 (2022).

2. Vogt, A.D. & Di Cera, E. Conformational selection or induced fit? A critical appraisal of the kinetic mechanism. *Biochemistry* **51**, 5894-902 (2012).

3. Lund, B.A. et al. The biological assembly of OXA-48 reveals a dimer interface with high charge complementarity and very high affinity. *FEBS J* **285**, 4214-4228 (2018).

4. King, D.T., King, A.M., Lal, S.M., Wright, G.D. & Strynadka, N.C. Molecular Mechanism of Avibactam-Mediated β-Lactamase Inhibition. *ACS Infect Dis* **1**, 175-84 (2015).

5. Fröhlich, C. et al. OXA-48-Mediated Ceftazidime-Avibactam Resistance Is Associated with Evolutionary Trade-Offs. *mSphere* **4**, e00024-19 (2019).

6. Docquier, J.D. et al. Crystal structure of the OXA-48 β-lactamase reveals mechanistic diversity among class D carbapenemases. *Chem Biol* **16**, 540-7 (2009).

7. Tooke, C.L. et al. Natural variants modify *Klebsiella pneumoniae* carbapenemase (KPC) acyl-enzyme conformational dynamics to extend antibiotic resistance. *J Biol Chem* **296**, 100126 (2021).

8. Patel, M.P. et al. The Drug-Resistant Variant P167S Expands the Substrate Profile of CTX-M β-Lactamases for Oxyimino-Cephalosporin Antibiotics by Enlarging the Active Site upon Acylation. *Biochemistry* **56**, 3443-3453 (2017).

9. Powers, R.A., Caselli, E., Focia, P.J., Prati, F. & Shoichet, B.K. Structures of ceftazidime and its transition-state analogue in complex with AmpC β-lactamase: implications for resistance mutations and inhibitor design. *Biochemistry* **40**, 9207-14 (2001).

10. Mitchell, J.M. et al. Structural basis of activity against aztreonam and extended spectrum cephalosporins for two carbapenem-hydrolyzing class D β-lactamases from *Acinetobacter baumannii*. *Biochemistry* **54**, 1976-87 (2015).
